# Modelling mortality and discharge of hospitalized stroke patients using a phase-type recovery model

**DOI:** 10.1007/s10729-018-9446-6

**Published:** 2018-05-01

**Authors:** Bruce Jones, Sally McClean, David Stanford

**Affiliations:** 1 Department of Statistical & Actuarial Sciences, Western University, London, Ontario N6A 5B7 Canada; 2 School of Computing & Information Engineering, Ulster University, Coleraine, BT52 1SA UK

**Keywords:** Stroke care, Length of stay, Phase type model, Conditional distribution

## Abstract

We model the length of in-patient hospital stays due to stroke and the mode of discharge using a phase-type stroke recovery model. The model allows for three different types of stroke: haemorrhagic (the most severe, caused by ruptured blood vessels that cause brain bleeding), cerebral infarction (less severe, caused by blood clots) and transient ischemic attack or TIA (the least severe, a mini-stroke caused by a temporary blood clot). A four-phase recovery process is used, where the initial phase depends on the type of stroke, and transition from one phase to the next depends on the age of the patient. There are three differing modes of absorption for this phase-type model: from a typical recovery phase, a patient may die (mode 1), be transferred to a nursing home (mode 2) or be discharged to the individual’s usual residence (mode 3). The first recovery phase is characterized by a very high rate of mortality and very low rates of discharge by the other two modes. The next two recovery phases have progressively lower mortality rates and higher mode 2 and 3 discharge rates. The fourth recovery phase is visited only by those who experience a very mild TIA, and they are discharged to home after a short stay. The novelty of our approach to phase representation is two-fold: first, it aligns the phases with labelled diagnosis states, representing stages of illness severity; second, the model allows us to obtain expressions for Key Performance Indicators that are of use to healthcare professionals. This allows us to use a backward estimation process where we leverage the fact that we know the phase of admission (the diagnosis), but not which phases are subsequently entered or when this happens; this strategy improves both computational efficiency and accuracy. The model has clear practical value as it yields length of stay distributions by age and type of stroke, which are useful in resource planning. Also, inclusion of the three modes of discharge permits analyses of outcomes.

## Background

Due to the debilitating nature of a stroke and complex makeup of the disease there is an urgent need for stochastic models that can be used for bed occupancy analysis, capacity planning, performance modeling and prediction, with a view to decreasing patient delays, better use of resources, and improved adherence to targets.

Modeling length-of-stay (LOS) in hospital is an important aspect of characterising patient stay in hospital and outcomes in the form of discharge destinations. We focus on using easily accessible administrative data routinely collected at discharge. Such data, which include information on patient date of birth, date of admission, diagnosis and discharge date, are not appropriate for patient prognostication but can rather be aimed towards supporting planning, service organization, and allocation of resources (see, for example, Shahani et al. [20], Faddy and McClean [5], Marshall and McClean [12] and McClean and Millard [14]). In such cases we are interested in the behavior of patient populations rather than individuals, with a focus on system wide planning.

Heterogeneity of patient pathways and LOS characteristics have been investigated by a number of authors. Such heterogeneity arises from a number of sources, for example, method of admission, diagnosis, severity of illness, age, gender, and treatment (see, for example, [6, 9, 10, 13]). Such covariates have previously been incorporated into phase-type models via conditional phase-type models by Marshall and McClean [12], a Coxian proportional hazards approach [5] and classification trees [9].

The philosophy behind our work stems from two facts. On the one hand, large amounts of data are usually required to validate analytical models, even if they are parsimonious in the number of parameters. On the other, often one is not free to gather as much data as one needs in order to determine the optimal number of parameters or stages for such a model. Rather, one often tends to obtain a dataset of a given size, without any option to enlarge it. It is in this context that we propose to bridge the gap by exploiting known physical properties underlying the problem being studied. In our case, the setting pertains to the recovery times for stroke patients of the three types most commonly encountered: haemorrhagic (the most severe, caused by ruptured blood vessels that cause brain bleeding), cerebral infarction (less severe, caused by blood clots) and transient ischemic attack, often abbreviated as TIA (the least severe, a mini-stroke caused by a temporary blood clot).

The novelty in what we present lies in the representation of the phases of the phase-type model as stages of illness severity, allowing the maximum likelihood estimation to be carried out using an efficient and accurate estimation process. Since our focus is on predicting the behavior of the patient population rather than individual outcomes, we can leverage data which only becomes available at discharge, e.g. discharge disposition; such information is commonly available in routinely collected patient data. Mathematical expressions and results for key performance indicators are presented for a phase-type model fitted to stroke patient data. There is further novelty in the application, as the model allows us to obtain expressions for Key Performance Indicators (KPIs) that are of use to healthcare professionals.

## Literature review

Analytic models have previously been developed for health services (see, for example, Fackrell [4] and Gunal and Pidd [2]). One of the first was a two-phase model (acute care and long-stay) introduced by Millard [18]. The model was fitted to length-of-stay (LOS) data from a number of different hospital and social services settings in Millard and McClean [15]. For stroke patients, a three-compartment approach (short stay, medium stay, long stay) was used in Vasilakis and Marshall [22] to estimate the number of patients in any state and their lengths-of-stay. McClean et al. [16, 17], Garg et al. [7] and Barton et al. [1] have generalized this to Markov models, based on the Coxian phase-type distributions, to help capacity planning within a stroke department. In addition, Gillespie et al. [8] and McClean et al. [17] have developed analytic cost models and applied them to a stroke service to assess the cost-effectiveness of the department.

Such phase-type or compartmental approaches to modelling patient sojourns in hospital, particularly for geriatric services and long-term conditions requiring long periods of rehabilitation, have been used for over 25 years (see, for example, Millard [18], Faddy and McClean [5, 6], and Xie et al. [23]). Typically, these approaches used only LOS calculated from admission and discharge dates. Subsequently a number of authors have tried to improve on the model fit and predictions by including available covariates, of which gender, age, diagnosis and discharge disposition are commonly available from standard healthcare datasets. Such variables have been shown to be statistically related to patient LOS and to improve modelling and prediction (see McClean et al. [16]). This resonates well with heterogeneity of patient pathways and LOS characteristics, in relation to such covariates, have previously been investigated by a number of authors. Such heterogeneity arises from a number of sources, for example, method of admission, diagnosis, severity of illness, age, gender, and treatment (see, for example, Faddy and McClean [6], Marshall and McClean [13], and Harper et al. [9]). Such covariates have previously been incorporated into phase-type models via conditional phase-type models by Marshall and McClean [12], a Coxian proportional hazards approach [5] and classification trees [9].

The stroke model presented in this paper was based on a 5-year retrospective dataset extracted from the Patient Administration System (PAS) and consisting of all patients admitted to the Belfast City Hospital (BCH) between 1 January 2003 and 31 December 2007 with a diagnosis of stroke. Data were available for date of admission, date of discharge, date of birth, gender, and discharge destination, and we have previously demonstrated relationships between these variables and LOS [16].

In the current paper we thus build on this work to explicitly represent the phases of the phase-type model as relating to the different types of stroke having occurred, with different illness severities. While such different diagnoses have different pathways in the acute phases, healthcare trajectories are likely to be similar in the recovery phases. In this way we incorporate diagnosis and discharge destination into the model definitions; the other covariates (here age and gender) are incorporated into the model as parametric functions with the expected properties. This approach has been used extensively in Survival Analysis generally, e.g. for Cox regression, and for LOS modelling in particular, (see, for example, Faddy and McClean [6] and McGrory et al. [11]).

## Stroke in-patient data

We here focus on incorporating age and diagnosis into a model of stroke patients in hospital. The modelling was based on five years’ retrospective data for patients admitted to the Belfast City Hospital with a diagnosis of stroke (cerebral hemorrhage, bleed in the brain; cerebral infarction, clot in the brain; transient ischemic attack, minor stroke; and unspecified or undetermined type of stroke). Data were obtained from the Patient Administration System, PAS (a computerized system that records patient activity relating to inpatients, outpatients, waiting lists, A&E and case note tracking). Data retrieved from the Belfast City Hospital PAS included age, diagnosis, LOS, and discharge destination. Diagnosis and age were previously shown to be highly significant with regard to LOS [16]. Our approach then models the patient journey through hospital as a phase-type model incorporating diagnosis and age. Summary information is shown in Table 1.

**Table 1:** Summary by type of stroke and mode of discharge

		Cerebral	
Mode of Discharge	Hemorrhagic	Infarction	TIA
Discharge Counts			
Death	65	125	13
Nursing Home	5	59	8
Usual Residence	69	432	389
Average lengths of stay (days)			
Death	18.3	34.6	37.5
Nursing Home	85.5	83.7	25.8
Usual Residence	51.3	31.9	8.2

The large number of deaths after a relatively short average length of stay confirms the high mortality rate of hemorrhagic stroke patients. Also, the large number of discharges to home after a very short average stay indicates the very mild nature of the TIA type of stroke.

## Model details

A probability distribution is said to be of “phase-type” if it can be shown to represent the time to absorption in a transient continuous-time Markov chain (see Neuts, 1981). The phases referred to are the successive times spent in the transient states, prior to absorption. The collection of phase-type distributions is commonly abbreviated as “PH distributions”, as we do hereafter.

We wish to underscore again that the model we present below involves states which are intended to have physical interpretations in the real world. Often when PH distributions are used to model lengths of stay for which an exponential distribution is inadequate, one aims to obtain the best possible fit for a given matrix order without imposing any structural restrictions in terms of associating the underlying states with a physical meaning.

Of course, there is no reason to anticipate that the ideal fit for a matrix of a given order will be sparse in terms of the number of parameters involved. Thus, there is an inherent disadvantage in seeking the best fit without regard to the associated number of parameters that would have to be estimated from the available data, and the corresponding impact in terms of reduced statistical power.

**Fig. 1:**
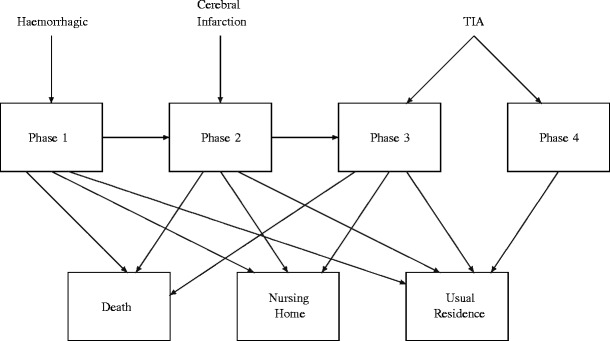
Initial state transition diagram

**Fig. 2:**
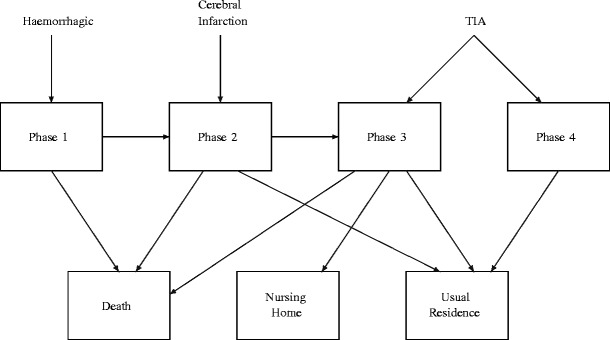
Revised state transition diagram

The model which we have decided upon strikes us as the best compromise between allowing for sufficient distinction of the various types of stroke to be considered, while maintaining a reasonable level of parsimony for parameter estimation. In fact, when we tried to make the model smaller as a check, there was a statistically significant reduction in the loglikelihood that was more than would be justified by the reduction in the number of parameters. Also, both our goodness of fit tests and the work of McClean et al. [16] show that phase-type models with this level of simplicity tend to fit this hospital length of stay data well. Ultimately, we wish to be able to distinguish paths based upon type of stroke incurred, and eventual disposition upon absorption. The model’s state transition diagram we initially considered is shown in Fig. 1, although we eventually decided upon an even more parsimonious model during the parameter estimation stage (see Fig. 2) by eliminating transitions from some transient recovery states to certain modes of discharge; this is discussed below in Section 6.

The structure presented in Fig. 1 was arrived at as follows. Since haemorrhagic strokes are generally the most debilitating, we anticipate three recovery phases for such patients. Those suffering from an infarction, being typically less severe in its degree of debilitation, are seen as passing through the latter two of the three recovery phases that haemorrhagic patients encounter. TIAs are collectively the least severe of all, but our study of the data revealed that they appear to comprise two distinct groups in terms of the duration of their recovery period. TIA patients experiencing longer recoveries are considered to pass through the last recovery phase of haemorrhagic patients and those with infarctions (namely, Phase 3), while there is a distinct state for very short TIA durations (Phase 4).

Transition rates for the model comprise some that depend upon the age and stroke type of the patient, and others which do not depend on age. Both for reasons of parsimony and for simplicity, we have chosen exponential forms for selected transition intensities and for the mixing probability for TIA patients; this ensures positive rates for the former and results in the interval (0,1) for the latter. We note that this does not mean we are using exponential distributions (with the corresponding memoryless property). Rather we are using an exponential functional form to incorporate covariates into the hazard functions for transition between states, rather in the way this is done in Cox regression.

For *i* = 1,2, let *λ*_*i*_(*x*) be the transition intensity from phase *i* to phase *i* + 1 for a patient who is age *x*, where *λ*_*i*_(*x*) = exp(*γ*_*i*_ + *β*_*i*_*x*). Also, let *p*(*x*) represent the probability that a TIA stroke patient age *x* is in recovery phase 4 upon admission to hospital (representing the less severe TIAs). Consequently, a TIA patient starts in phase 3 with probability 1 − *p*(*x*). We assume that *p*(*x*) = exp{−exp(*𝜃*_0_ + *𝜃*_1_*x*)}. The exponential functions used in modelling *λ*_*i*_(*x*) and *p*(*x*) are fairly standard, and ensure that their values are constrained to the required ranges. These functions arise when using the log link and complementary log-log link functions in generalized linear models (see [3]). As regards the parameters which do not depend upon age, for *i* = 1,…,4,*μ*_*i*_ denotes the mortality rate from phase *i*,*ν*_*i*_ the rate of discharge to a nursing home from phase *i*, and *ρ*_*i*_ the rate of discharge to the usual residence from phase *i*. As indicated in Fig. 1, it is assumed that *μ*_4_ = *ν*_4_ = 0.

We have consciously decided not to have the rates of mortality and discharge to nursing home and usual residence depend on age. For mortality, the age effect is dominated by the extra mortality that results from the stroke, the severity of which depends on the phase of the patient. This is influenced by the type of stroke and the time since admission. It has been assumed, though, that the probability of a more severe TIA and the first two rates of recovery (progression through the phases) do depend on age. This creates age effects in how fast the stroke related extra mortality diminishes as well as how the rates of discharge to nursing home and usual residence change with time since admission. While we tested for gender differences, we found them to be insignificant.

The infinitesimal generator matrix **Q** for our model can be written as

1 $$ \textbf{Q} = \left( \begin{array}{cc} \textbf{T} &t_{A} \\ \textbf{0}_{AT} &\textbf{0}_{AA} \end{array}\right) $$

where **T** = (*t*_*ij*_) is a 4 × 4 matrix containing transition rates among the transient states, given by

2 $$ \textbf{T} = \left( \begin{array}{cccc} -{\Lambda}_{1}(x) & \lambda_{1}(x) & 0 & 0\\ 0 & -{\Lambda}_{2}(x) & \lambda_{2}(x) & 0\\ 0 & 0 & -{\Lambda}_{3} & 0\\ 0 & 0 & 0 & -{\Lambda}_{4} \end{array}\right) $$

for Λ_*i*_(*x*) = *λ*_*i*_(*x*) + *μ*_*i*_ + *ν*_*i*_ + *ρ*_*i*_;*i* = 1,2 and Λ_*i*_ = *μ*_*i*_ + *ν*_*i*_ + *ρ*_*i*_;*i* = 3,4. In like fashion, **t**_*A*_ = (*t*_*ij*_);*i* = 1,2,3,4;*j* = 5,6,7 is a 4 × 3 matrix containing absorption rates for the various destination modes (death, nursing home, and usual residence, respectively), given by

3 $$ \textbf{t}_{A} = \left( \begin{array}{ccc} \mu_{1} & \nu_{1}^{*} & \rho_{1}^{*} \\ \mu_{2} & \nu_{2}^{*} & \rho_{2}\\ \mu_{3} & \nu_{3} & \rho_{3}\\ \mu_{4} & \nu_{4} & \rho_{4} \end{array}\right). $$

In Eq. 3, three of the rates have been appended with asterisks. These identify the parameters from the original model that we ultimately treated as zero as a result of the initial parameter estimation step (see Section 6). Finally, **0**_*AT*_ and **0**_*AA*_ are null matrices of appropriate dimensions; **0** is a null column vector. These elements satisfy the conditions *t*_*ii*_ < 0, for *i* = 1,…,7,*t*_*ij*_ ≥ 0, for *i*≠*j*. **T** and **t**_*A*_ satisfy **t**_*A*_**1**_3_ = −**T 1**_4_, where **1**_*n*_ is an *n*-dimensional column vector of ones.

Letting *X* denote the stroke length of stay random variable, the event {*X* > *y*} implies that recovery is ongoing at time *y*. Thus, for a given initial distribution of recovery phases ***α***, the probability density function of *X* is given by

4 $$ f_{X}(y\,|\boldsymbol{\alpha},\textbf{T}) =\boldsymbol{\alpha}^{\prime}\,\exp({\textbf{T}y})\,\textbf{t}_{A}\textbf{1}_{3}\,, \,\, y \geq 0\,. $$

(These and other matrix-based operations are justified in the light of Neuts [19], pp. 45-46). In the foregoing, the exponential of a square matrix **A** is given by $\exp ({\textbf {A}}) = {\sum }_{k = 0}^{\infty } {{{\textbf {A}^{k}}\over {k!}}}$. The partial density functions by discharge mode can similarly be obtained. Integrating (4), the distribution function of *X* is

5 $$\begin{array}{@{}rcl@{}} F_{X}(y\,|\,\boldsymbol{\alpha},\textbf{T}) &=& 1-\boldsymbol{\alpha^{\prime}}\,\exp({\textbf{T}y})\,(-\textbf{T})^{-1}\textbf{t}_{A}\textbf{1}_{3} \\ &=& 1-\boldsymbol{\alpha^{\prime}}\,\exp({\textbf{T}y})\,\textbf{1}_{4}\,, \,\, y \geq 0, \end{array} $$

The 4 × 3 matrix **P** = (−**T**)^− 1^**t**_*A*_ is of interest in its own right, and can be interpreted as the probability of being absorbed into the various discharge modes (death, nursing home, or regular residence), starting from the various recovery phases. This is equivalent to similar expressions previously given in McClean et al. [16].

We are also interested in length of stay by discharge mode, so that we can obtain the conditional distributions by dividing by the ultimate probability of absorption into the appropriate states.

## Conditional mean durations and distributions

One of the merits of the phase type modelling approach is that it lends itself quite readily to the determination of expectations and distributions based upon a variety of conditions. The overall mean duration of a stroke hospitalisation, considering all possible types and outcomes, is given by

6 $$ E\{X\} =\boldsymbol{\alpha}^{\prime}(-T^{-1})\textbf{1}_{4}. $$

This mean comprises three distinct pieces. The contribution due to patients who ultimately die is given by

7 $$ E\{X\ I(\textbf{Death})\} =\boldsymbol{\alpha}^{\prime}(-T^{-1})\textbf{P}_{1} $$

where *I*(**Death**) denotes the indicator function for the event that a death occurs, and **P**_1_ denotes the first column of the matrix **P**. Therefore, the conditional expectation for the length of hospitalisation, given that a patient dies, is given by

8 $$ E\{X|\textbf{Death}\} \,=\, E\{X\ I(\textbf{Death})\}/P(\textbf{Death}) \!=\boldsymbol{\alpha}^{\prime}(\!-T^{-1})\textbf{P}_{1}/(\boldsymbol{\alpha}^{\prime}\textbf{P}_{1}). $$

The mean hospitalisation duration for patients who return to their own home, as well as that for patients who go to a nursing home, can be similarly obtained.

Another way in which the model can be employed for useful inferences is to determine residual lengths of stay for patients who have already been hospitalised for a given time, say of duration *y*. The probability of such an event is

9 $$ P(X>y) = \boldsymbol{\alpha}^{\prime}\,\exp({\textbf{T}y})\textbf{1}_{4} $$

and the mean residual hosptialisation for such patients is given by

10 $$ E\{X-y|X>y\} = \boldsymbol{\alpha}^{\prime}\,\exp({\textbf{T}y})(-T^{-1})\textbf{1}_{4}/(\boldsymbol{\alpha}^{\prime}\,\exp({\textbf{T}y})\textbf{1}_{4})\,, y \!> 0. $$

Finally, we consider the impact of hospitalisation duration on the relative likelihoods of the various possible destinations for the given patient. Intuitively, we expect that the longer a stroke patient is hospitalised, the less risk there is that the patient will die. Mathematically, the phase type model predicts that the impact of a hospitalisation of length *y* on the various possible discharge destinations is given by the 4 × 3 matrix

11 $$ \textbf{P}(y) = \boldsymbol{\alpha}^{\prime}\,\exp({\textbf{T}y})\textbf{P}/(\boldsymbol{\alpha}^{\prime}\,\exp({\textbf{T}y})\textbf{1}_{4})\,, y > 0. $$

## Estimation

The total number of parameters to be estimated is 16. We employed maximum likelihood estimation. The likelihood function is given by the product over all 1,234 stroke patients of the likelihood contribution for that patient. The likelihood contribution for a given patient is proportional to the model probability or probability density associated with our observation for that patient.

Phase-type models have previously been used for patient modelling by a number of authors, as described in Section 1. However, since the phases are usually latent states of the underlying Markov model, parameter estimation can be challenging, with high numbers of parameters and large variances of parameter estimates. The novelty here is that by aligning the phases with labelled diagnosis states, which represent stages of illness severity, the estimation process reduces to a problem of semi-supervised learning where we know the phase of admission (the diagnosis), but not which phases are subsequently entered or when. Our current estimation strategy allows us to use maximum likelihood in an iterative manner moving backwards through the phases, where we progress from estimating transition parameters for the least severe patients to the most severe. At each iteration, parameters relating to transitions and data from patients entering into that phase are added to the estimation process while previously estimated parameters, pertaining to less severe phases, are left unchanged if no further information has been provided. As such, we are able to use the data on phase of admission to its best use, as well as patient duration in hospital, thus improving both computational efficiency and accuracy.

Let *L*_*j*_ represent the likelihood contribution for patient *j*. If patient *j* is a TIA patient, then

12 $$\begin{array}{@{}rcl@{}} L_{j} &=& (1-p(x_{j}))\exp\{-(\mu_{3}+\nu_{3}+\rho_{3})\,s_{j}\}\,\mu_{3}^{\delta_{j}^{\mu}}\, \nu_{3}^{\delta_{j}^{\nu}}\,\rho_{3}^{\delta_{j}^{\rho}}\\&& + p(x_{j})\exp\{-\rho_{4}\,s_{j}\}\, \mu_{4}^{\delta_{j}^{\mu}} \, \nu_{4}^{\delta_{j}^{\nu}} \,\rho_{4}^{\delta_{j}^{\rho}}, \end{array} $$

where *s*_*j*_ is the length of stay of patient *j*,*x*_*j*_ is the age at admission of patient $j, \delta _{j}^{\mu }$ is 1 if patient *j* died and 0 otherwise, $\delta _{j}^{\nu }$ is 1 if patient *j* was discharged to a private nursing home and 0 otherwise, and $\delta _{j}^{\rho }$ is 1 if patient *j* was discharged to his/her usual residence and 0 otherwise.

In interpreting equation (12), recall that *μ*_4_ = 0 and *ν*_4_ = 0; furthermore, we treat 0^0^ as 1. Therefore, for a TIA patient who died ($\delta _{j}^{\mu }= 1$) or was discharged to a nursing home ($\delta _{j}^{\nu }= 1$), the second term on the right-hand side of Eq. 12 is zero. Hence, *L*_*j*_ is the probability of a more severe TIA times the density associated with a stay of length *s*_*j*_ ending in death (if $\delta _{j}^{\mu }= 1$) or discharge to a nursing home (if $\delta _{j}^{\nu }= 1$). For a patient who was discharged to his/her usual residence ($\delta _{j}^{\rho }= 1$), *L*_*j*_ is the sum of two terms: the probability of a more severe TIA times the conditional density of a stay of length *s*_*j*_ ending in discharge to usual residence (given it is a more severe TIA), and the probability of a less severe TIA times the conditional density of a stay of length *s*_*j*_ ending in discharge to usual residence (given it is a less severe TIA).

The likelihood contributions for the other two types of stroke are more complicated. To simplify the expressions, let

$$r_{1j} = \mu_{1}+\nu_{1}+\rho_{1}+\lambda_{1}(x_{j}), $$

$$r_{2j} = \mu_{2}+\nu_{2}+\rho_{2}+\lambda_{2}(x_{j}), $$

where *x*_*j*_ denotes the age upon admission and

$$r_{3} = \mu_{3}+\nu_{3}+\rho_{3}. $$

If patient *j* is a Cerebral Infarction patient, then

13 $$\begin{array}{@{}rcl@{}} L_{j} \!&=& \exp\{-r_{2j}\,s_{j}\}\,\mu_{2}^{\delta_{j}^{\mu}}\,\nu_{2}^{\delta_{j}^{\nu}}\,\rho_{2}^{\delta_{j}^{\rho}}\\ &&+ \lambda_{2}(x_{j})\frac{\exp\{-r_{3}\,s_{j}\}\,-\,\exp\{-r_{2j}\,s_{j}\}}{r_{2j}-r_{3}}\mu_{3}^{\delta_{j}^{\mu}}\, \nu_{3}^{\delta_{j}^{\nu}}\,\rho_{3}^{\delta_{j}^{\rho}}. \end{array} $$

The first term above corresponds to the density associated with a CI patient stay in phase 2 equal to *s*_*j*_ before a direct transition is made to the death state (if $\delta _{j}^{\mu }= 1$) or the usual residence state (if $\delta _{j}^{\rho }= 1$). [Since *ν*_2_ = 0, direct transition to a nursing home is not allowed.] The second term is the density associated with a CI patient moving from phase 2 to phase 3 and then dying (if $\delta _{j}^{\mu }= 1$), being discharged to a nursing home (if $\delta _{j}^{\nu }= 1$) or being discharged to usual residence (if $\delta _{j}^{\rho }= 1$) after a total stay of *s*_*j*_.

If patient *j* is a Haemorrhagic stroke patient, then

14 $$\begin{array}{@{}rcl@{}} L_{j} & = & \exp\{-r_{1j}\,s_{j}\}\,\mu_{1}^{\delta_{j}^{\mu}}\,\nu_{1}^{\delta_{j}^{\nu}}\,\rho_{1}^{\delta_{j}^{\rho}}+ \lambda_{1}(x_{j})\, \frac{\exp\{-r_{2j}\,s_{j}\}-\exp\{-r_{1j}\,s_{j}\}}{r_{1j}-r_{2j}}\,\mu_{2}^{\delta_{j}^{\mu}}\, \nu_{2}^{\delta_{j}^{\nu}}\,\rho_{2}^{\delta_{j}^{\rho}} \\ & & +\frac{\lambda_{1}(x_{j})\,\lambda_{2}(x_{j})}{r_{1j}-r_{2j}}\, \left( \frac{\exp\{-r_{3}\,s_{j}\}-\exp\{-r_{2j}\,s_{j}\}}{r_{2j}-r_{3}}- \frac{\exp\{-r_{3}\,s_{j}\}-\exp\{-r_{1j}\,s_{j}\}}{r_{1j}-r_{3}}\right)\\ & & \times\mu_{3}^{\delta_{j}^{\mu}}\, \nu_{3}^{\delta_{j}^{\nu}}\,\rho_{3}^{\delta_{j}^{\rho}}. \end{array} $$

The interpretation of Eq. 14 is similar to Eq. 13, except that for Haemorrhagic stroke patients, *L*_*j*_ involves three terms, reflecting hospital stays ending upon transition from phase 1, phase 2 or phase 3.

We construct the log-likelihood function by summing the logarithms of the likelihood contributions, and this function is maximized with respect to the 16 parameters to obtain the estimates of these parameters.

It is challenging to maximize a function of 16 parameters without good starting values. We are, however, able to obtain reasonable starting values by first considering subsets of the data based on type of stroke. Referring to the discharge counts in Table 1, we see that the TIA data provide mostly information about *ρ*_3_ and *ρ*_4_, and all of the information about *ρ*_4_. The Cerebral Infarction data provide additional information about *ρ*_3_ and most of the information about *μ*_3_, *ν*_3_, *μ*_2_, *ν*_2_ and *ρ*_2_. Finally, the Haemorrhagic stroke data provide additional information about *μ*_2_ and *ρ*_2_ as well as all of the information about *μ*_1_, *ν*_1_ and *ρ*_1_. It is not uncommon in health care data for specific subsets of the data to provide more information about certain patient pathways, and this can be taken advantage of as we have here.

We began by using the TIA data only to obtain preliminary estimates of *𝜃*_0_, *𝜃*_1_, *μ*_3_, *ν*_3_, *ρ*_3_ and *ρ*_4_. With these parameters held fixed, we then used the Cerebral Infarction data only to obtain preliminary estimates of *γ*_2_, *β*_2_, *μ*_2_, *ν*_2_ and *ρ*_2_. Combining the TIA and Cerebral Infarction data, we updated the preliminary estimates of the 11 parameters. Next, with these parameters held fixed, we used the Haemorrhagic stroke data only to obtain preliminary estimates of *γ*_1_, *β*_1_, *μ*_1_, *ν*_1_ and *ρ*_1_. Finally, the preliminary estimates were used as starting values to estimate all 16 parameters using all of the data combined.

The results of the initial estimation process showed that three of the initial parameters (namely, *ν*_1_, *ν*_2_ and *ρ*_1_) were not meaningfully different from zero, as the associated *p*-values were all in excess of 90 percent. We therefore revised the original state transition diagrams to eliminate these transitions. These eliminations also make sense, in that Phase 1 pertains to seriously ill patients, for whom any sort of discharge other than by death is unrealistic. Similarly, there would be no reason to transfer patients from Phase 2 to a nursing home without availing of the normal amount of recovery time provided by Phase 3. The resulting diagram appears in Fig. 2.

After setting *ν*_1_, *ν*_2_ and *ρ*_1_ to 0, we obtained the parameter estimates shown in Table 2. An asymptotic covariance matrix is obtained as the inverse of the observed information matrix evaluated at the maximum likelihood estimates. The latter matrix is found as a byproduct of the numerical method used to maximize the log-likelihood function. The standard error estimates shown in Table 2 are the square roots of the diagonal elements of the asymptotic covariance matrix. The Z-statistics are simply the parameter estimates divided by the standard errors, and each can be used to test the hypothesis that the corresponding parameter equals 0. The p-values, based on asymptotic normality of the parameter estimators, indicate rather strong evidence against the null hypothesis in each case.

**Table 2:** Parameter estimates

Parameter	Estimate	Std Error	Z-Stat	p-value
*γ*_1_	6.63570	1.21893	5.44388	0.00000
*β*_1_	− 0.03652	0.01631	− 2.23902	0.02515
*γ*_2_	− 3.06931	1.22697	− 2.50153	0.01237
*β*_2_	0.07153	0.01667	4.29057	0.00002
*𝜃*_0_	− 8.66118	1.48644	− 5.82680	0.00000
*𝜃*_1_	0.08801	0.01828	4.81391	0.00000
*μ*_1_	22.10156	4.95434	4.46105	0.00001
*μ*_2_	2.48820	0.37993	6.54912	0.00000
*μ*_3_	1.56162	0.20294	7.69509	0.00000
*ν*_3_	1.27849	0.17391	7.35165	0.00000
*ρ*_2_	11.76860	0.99634	11.81180	0.00000
*ρ*_3_	3.41989	0.38393	8.90762	0.00000
*ρ*_4_	63.92514	4.11394	15.53865	0.00000

In order to check the fit of our model, we considered comparisons of nonparametric estimates of the cumulative intensity functions for the different modes of discharge with estimates of the cumulative intensity function based on our fitted model. Since the latter estimates depend on age at admission, we examined nonparametric estimates for three age groups. Specifically, we plotted the well-known Nelson-Åalen estimates of the cumulative intensity function for each of the age intervals [60,70),[70,80) and [80,90) for each type of stroke and each mode of discharge for which we have a meaningful number of discharges. These plots are shown in Figs. 6, 7, 8, 9, 10 and 11 in the Appendix. Along with the Nelson-Aalen estimates in each graph, we also plotted the fitted model cumulative intensity function for the endpoints and midpoint of the age interval.

For most of the graphs, we observe a reasonably good fit. The fitted model estimates conform fairly well with the nonparametric estimates, and when the model estimates are not very close, they tend to stay within the 95 percent confidence limits for most length of stay values.

## Results

At this point, we turn our attention from parameter estimation to some examples of what the model can be used for. The first such measure we present indicates, for three ages, the likelihoods of the possible destinations upon discharge for each initial recovery phase. The results presented in Table 3 indicate the relative likelihoods as a percentage for an individual aged 65, 75, and 85, respectively. We observe that as patients age, more Haemorrhagic patients tend to die and fewer are discharged to their usual residence. Relatively speaking, there is much less impact upon the Infarction patients, with a very small increased likelihood of mortality, accompanied by a larger likelihood of going to a nursing home. These relative likelihoods are in keeping, qualitatively speaking, with what one might anticipate from the relative severity of these two types of stroke. For TIA patients, we see that the likelihoods of death and discharge to nursing home increase with age, and the likelihood of discharge to usual residence decreases with age. This is expected, as the probability of a more severe TIA, for which the patient begins in phase 3, is an increasing function of age.

**Table 3:** Ultimate destination percentage by age and type of stroke

	Death	Nursing home	Usual residence
Age 65			
Haemorrhagic	38.5	4.0	57.5
Cerebral infarction	19.4	5.2	75.5
TIA	1.3	1.0	97.7
Age 75			
Haemorrhagic	45.1	5.8	49.1
Cerebral infarction	20.5	8.4	71.1
TIA	3.0	2.4	94.6
Age 85			
Haemorrhagic	52.5	7.3	40.1
Cerebral infarction	21.9	12.0	66.1
TIA	6.6	5.4	88.0

**Fig. 3:**
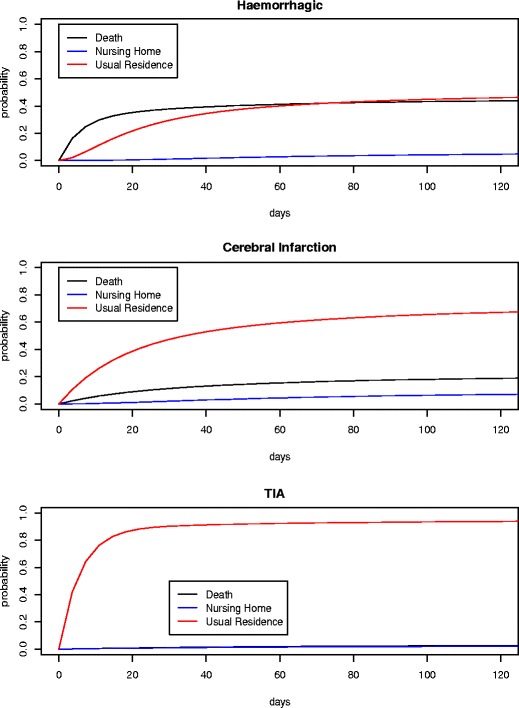
Cumulative probability of discharge by type of stroke and destination

Figure 3 presents the cumulative probability of discharge as a function of the type of stroke for each of the modes of discharge: death, nursing home, and usual residence (i.e. home). In the case of haemorrhagic strokes (top panel), we see that the deaths that occur tend to happen quickly, with most of them having happened within the first 10 days since onset of the stroke. In contrast, the discharges to the usual residence take much longer, as an extended period is needed to pass through the corresponding recovery phases before being discharged home. As there are very few cases on record of discharge to a nursing home in the event of haemorrhagic stroke, little can be inferred, other than the fact that it tends to take a lot of time.

The middle panel of Fig. 3 reveals that the chance of death is markedly reduced in the case of cerebral infarctions, and that those deaths that do occur tend to happen over the course of the stay (perhaps due to onset of a further stroke event while recovering, as the data does not record such distinctions). The likelihood of discharge to a nursing home is also greater than either of the other types of stroke, but even in this case, death is about twice as likely as discharge to nursing home.

The story in the case of TIAs is rather straightforward, with in excess of ninety percent of patients being discharged to their usual residence. Once again, from a medical perspective, one might suspect that those few patients who die or are discharged to a nursing home are likely to have incurred another stroke, but as our data does not provide this information, we cannot make such a conclusion from the model.

**Fig. 4:**
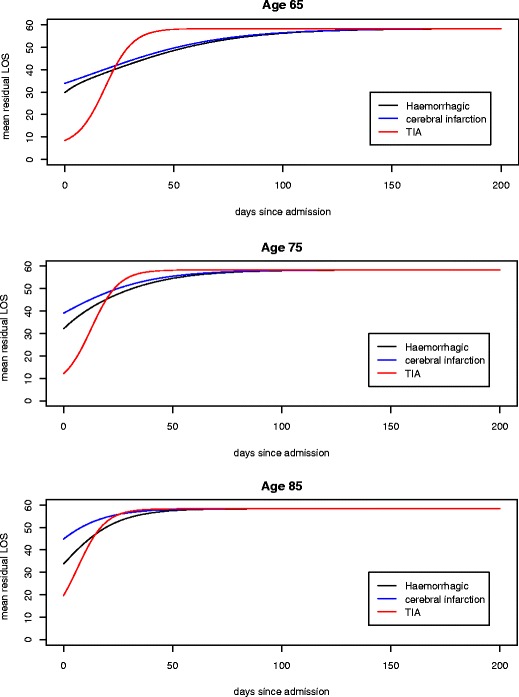
Mean residual length of stay by age and type of stroke

Figure 4 displays how the mean residual length of stay (LOS) depends upon the patient’s age and type of stroke, as a function of their incurred LOS. For patients of age 65, there is little difference between the curves for Haemorrhagic and Infarction patients, and what little difference there is tends to diminish quickly. We observe that the mean LOS for Haemorrhagic patients is smaller than that for Infarction patients; this reflects the increased mortality of the former relative to the latter. We observe an increased mean residual stay for TIA patients; this reflects the fact that after a few days, all of the quick recoveries have occurred, and the pertinent patients have been been discharged. In the limit, all curves tend to a mean residual stay of about 60 days, indicating that all patients are virtually assured of being in the last common recovery stage.

The situation for patients of ages 75 and 85 is much the same, except that we note a larger initial discrepancy between Haemorrhagic and Infarction patients, reflecting increased likelihood of death for Haemorrhagic patients at older ages. The TIA curve rises more quickly to its limiting value, reflecting a larger number of patients suffering the more severe type of TIA.

**Fig. 5:**
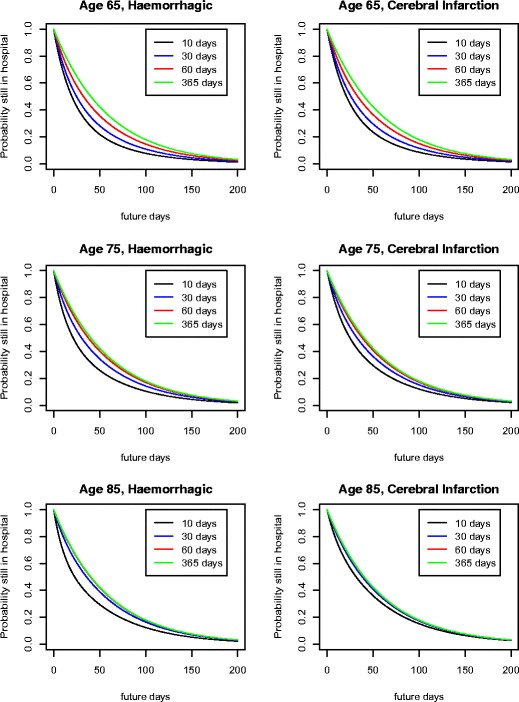
Probability of remaining in hospital by age, type of stroke, and length of stay to date

**Fig. 6:**
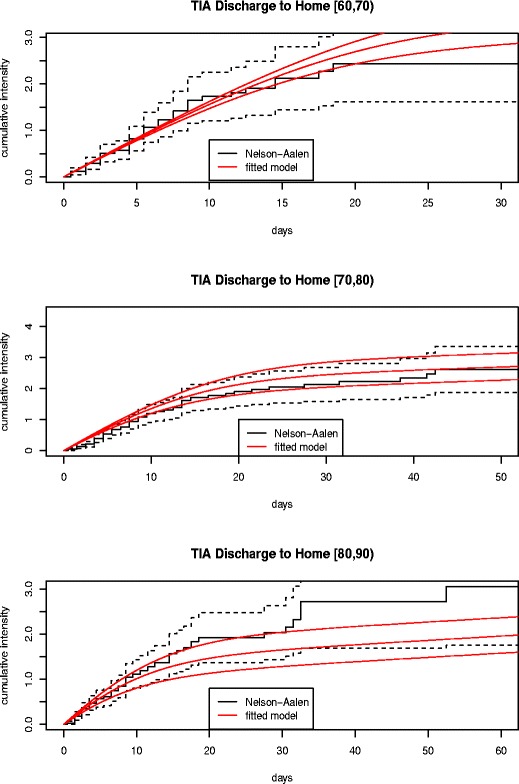
Nelson-Aalen & fitted model estimates of the cumulative intensity function for TIA patient discharges to their usual residence. Nelson-Aalen estimates appear in black along with approximate 95% confidence limits (dashed). The red curves represent the fitted cumulative intensity function at the endpoint ages and middle age

**Fig. 7:**
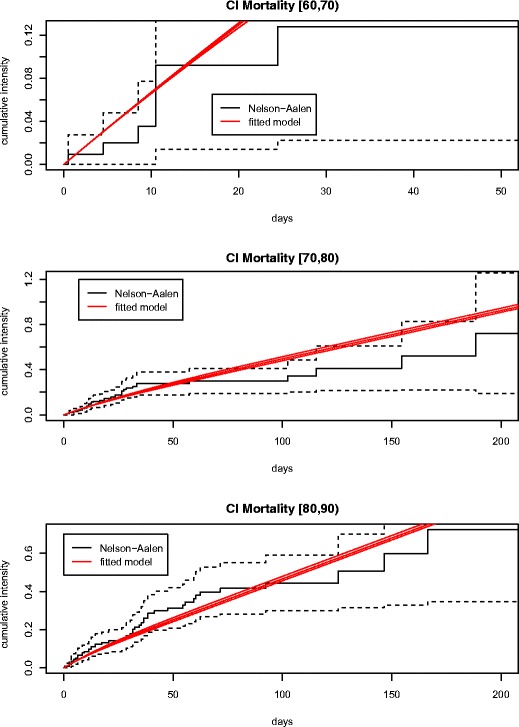
Nelson-Aalen & fitted model estimates of the cumulative intensity function for Cerebral Infarction patient deaths. Nelson-Aalen estimates appear in black along with approximate 95% confidence limits (dashed). The red curves represent the fitted cumulative intensity function at the endpoint ages and middle age

**Fig. 8:**
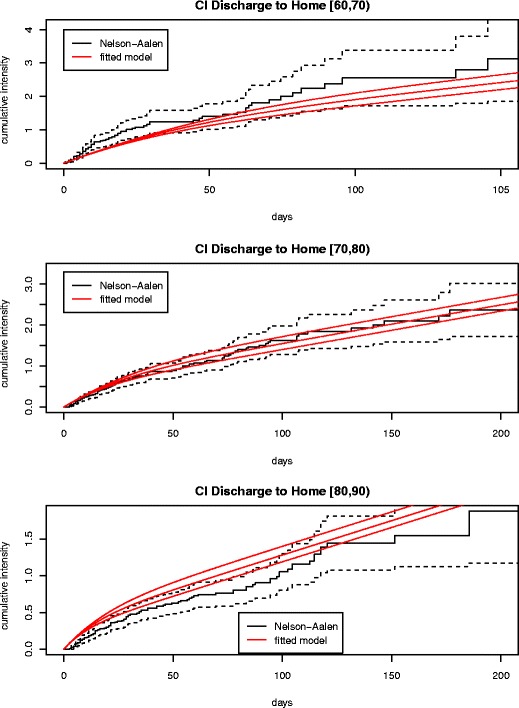
Nelson-Aalen & fitted model estimates of the cumulative intensity function for Cerebral Infarction patient discharges to their usual residence. Nelson-Aalen estimates appear in black along with approximate 95% confidence limits (dashed). The red curves represent the fitted cumulative intensity function at the endpoint ages and middle age

**Fig. 9:**
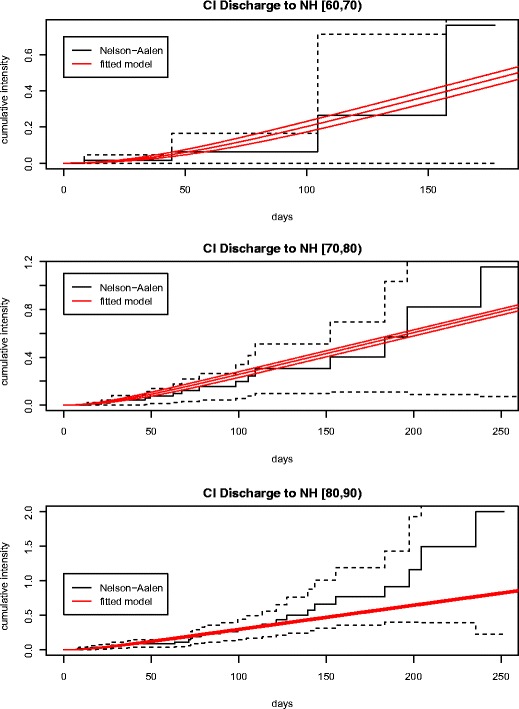
Nelson-Aalen & fitted model estimates of the cumulative intensity function for Cerebral Infarction patient discharges to a private nursing home. Nelson-Aalen estimates appear in black along with approximate 95% confidence limits (dashed). The red curves represent the fitted cumulative intensity function at the endpoint ages and middle age

**Fig. 10:**
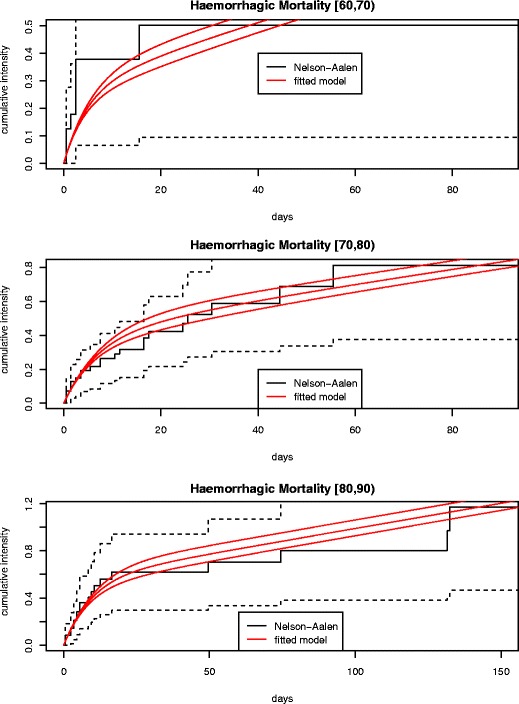
Nelson-Aalen & fitted model estimates of the cumulative intensity function for Haemorrhagic patient deaths. Nelson-Aalen estimates appear in black along with approximate 95% confidence limits (dashed). The red curves represent the fitted cumulative intensity function at the endpoint ages and middle age

**Fig. 11:**
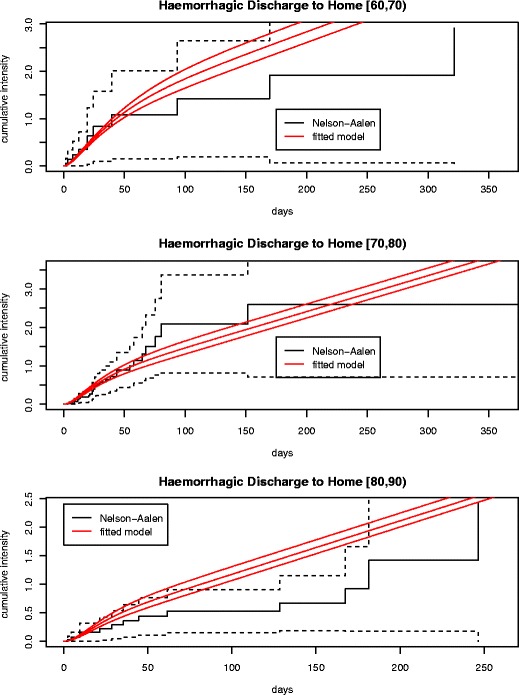
Nelson-Aalen & fitted model estimates of the cumulative intensity function for Haemorrhagic patient discharges to their usual residence. Nelson-Aalen estimates appear in black along with approximate 95% confidence limits (dashed). The red curves represent the fitted cumulative intensity function at the endpoint ages and middle age

Figure 5 expands upon the foregoing analysis by presenting the probabilities of various lengths of further stay for Haemorrhagic and Infarction patients as a function of the incurred stay. As expected, the longer a patient has stayed, the longer we expect the residual stay to be, since the quicker recoveries have already been discharged. For a patient of age 65, we still observe a meaningful difference in terms of the length of further stays for patients who have already stayed two months versus those who have stayed one year. However, this distinction has virtually disappeared for patients of age 75, and by the time the patient has reached 85 years of age, there is virtually no difference even after one month of stay.

Table 4 presents the mean lengths of stay by age, type of stroke, and ultimate destination. We note that the Haemorrhagic patients who die tend to do so within a few weeks of admission. A comparison of the overall mean lengths of stay reveals a shorter average stay for Haemorrhagic patients than for Infarction patients, and this only makes sense by accounting for the higher mortality rate in the first recovery phase. This pattern is observed for all three age groups, where older patients tend to stay in hospital longer, irrespective of the diagnosis, with the exception of the TIA patients whose stroke seems to be so minor that age is not a factor. In all cases patients who died in hospital had a shorter stay in hospital than patients who were discharged to home, and this latter group of patients had a shorter stay in hospital than patients who were discharged to a private nursing home. This pattern is as expected, as the first group are very ill and do not survive for long, whereas we would expect the last group to require the longest recovery and rehabilitation period in hospital.

**Table 4:** Mean length of stay (Days) by age, type of stroke and ultimate destination

	Death	Nursing home	Usual residence	All
Age 65				
Haemorrhagic	18.6	81.3	33.7	29.8
Cerebral infarction	38.2	77.4	29.8	33.9
TIA	58.3	58.3	7.2	8.4
Age 75				
Haemorrhagic	19.0	78.5	38.6	32.1
Cerebral infarction	44.2	73.4	33.5	39.0
TIA	58.3	58.3	9.3	12.0
Age 85				
Haemorrhagic	19.0	75.4	45.3	33.7
Cerebral infarction	49.7	68.9	38.9	44.8
TIA	58.3	58.3	14.3	19.6

## Discussion

Overall, modelling can be used to characterize the whole system of stroke patient care and the associated pathways, integrating hospital and community services to provide tools for describing current services, assessing the impact of proposed changes, and predicting resource requirements in future scenarios. Our current focus is on developing models that use routinely available hospital discharge data to describe patient admissions, movements through hospital, and discharge to, inter alia, community services, such as nursing homes. By describing movements of patients along pathways, a model can be used to facilitate performance modelling, bed occupancy analysis, capacity planning, and prediction of patient numbers in different components of the overall care system. By using a model to quantify resource consumption, and costs of such proposed interventions, we can compare different solutions and determine optimal strategies for the whole integrated patient care system. Within stroke patient care, there are many possible interventions which straddle hospital and community services, which often operate separate budgets. However, by utilizing planning tools that encapsulate the whole system we can determine optimal integrated strategies, to the overall benefit of the service and the patient.

For such models to be effective, a robust estimation process and thorough evaluation is essential. However, to achieve these goals a large amount of data is required even to estimate a small number of parameters. This is the case in our current study where the variables we consider are LOS, discharge destination, age, and diagnosis, as extracted from the, routinely available, discharge summary data. In addition, a number of the variables are continuous (LOS and age), potentially increasing the number of parameters to estimate, even when we employ models. On the other hand, the LOS distributions are highly diverse, varying from hospital to hospital according to patient management strategies and catchments. In addition, the situation is typically highly dynamic with management practices changing rapidly. These sampling issues have an impact on validation as, with such inherently limited sample sizes, we cannot afford to perform model testing based on hold-out samples; as a result, we have, in this paper, validated the model using all the data for estimation and Z-tests to determine if the parameters are significantly different from zero. This strategy is primarily proposed in this paper because it is difficult to get enough data so that the LOS distribution is stable, as discussed. As a result, the model should only be used for short-term predictions and to identify the way in which the system is changing.

Modelling is a particularly useful approach for understanding stroke patient pathways. Due to the potentially serious and debilitating nature of stroke, many patients have a prolonged LOS in hospital, often considered to be an inefficient use of resources, as well as a distressing time for the patients and their families. Using modelling, we can create scenarios that explore the potential benefits of interventions such as early discharge schemes, drug therapy e.g. thrombolysis (clot busting drugs), surgery and CT scanning, that impact on crucial aspects such as LOS in hospital, destination on discharge, and quality of life subsequent to discharge. Overall LOS is a key performance indicator for hospital services, and it is therefore useful to be able to analyse the different components of LOS and assess the impact of key interventions, in terms of their impact on LOS for different hospital phases, diagnoses and demographic profiles. A particular aspect of this is the influence of discharge delays on overall LOS where, due to the lack of availability of a suitable placement, patients may undergo an extended stay in hospital. In addition, delayed discharge not only negatively affects the hospital performance metrics, but also has other serious consequences for the healthcare system such as bed blocking and potentially a negative effect on patients’ health and quality of life. In the next few paragraphs we describe how our initial model can be used to accommodate such interventions by modifying the rates or structure of the model. Possible mechanisms follow for early discharge, based on rate modification, and thrombolysis, based on structure modification.

For early discharge we have used a previous (much less parsimonious) phase-type model fitted to the same data as used in the current paper to assess the effect of increasing the delay in discharge to private nursing home (PNH) on the overall LOS in hospital [16]. A systematic increase in the LOS of patients discharged to PNH (achieved by modifying the rates of discharge to PNH appropriately) resulted in an increase of overall average LOS in hospital, as expected. Results for delayed discharge were further explored in McClean et al. [16], where the relationship between average overall LOS and delay in discharge to PNH, when the patients have to queue for discharge, was explored. Here, the effect of the queue on overall LOS was much more dramatic than was the case without queueing, as expected. For our current model, a similar approach is likely to yield similar results and provide insights into the impact of potential service improvements.

The previous model introduced in McClean et al. [16] was also used as a basis for assessing the effective of thrombolysis on costs and quality of life [8]. For those patients who receive thrombolysis, the LOS in acute care has been estimated to be reduced by 2 days [21]. Also it is the case that only patients with a Cerebral Infarction can benefit from thrombolysis. We therefore introduce a new state (Phase 2*) to the model, to represent initial hospitalization for patients with Cerebral Infarction who have been thrombolysed. There is then a probability of thrombolysis and 1- or not being thrombolysed, on entry to this state. All transitions from Phase 2* are as for Phase 2 except that for each possible transition the rate is adjusted to reduce the average LOS in phase 2* by 2 days. The impact of these interventions on patients’ LOS can then be found as for the primary model, as presented in Section 4 (the theory) and Section 5 (the results). Costs can be associated with these stays as can quality-adjusted life years (QALYs); see for example Barton et al. [1].

## Conclusions and further work

We have developed a phase-type modelling approach with particular applicability to stroke patient care. Since in most cases, there are multiple outcomes for patients, such as discharge to normal residence, nursing home, or death, we have presented a phase-type model with a number of absorbing states. In terms of modelling stroke patients, we are particularly interested in discharges to private nursing homes, which may be responsible for bottlenecks, and resulting delayed discharge. Such delays can have a significant effect on expected LOS in hospital, which is a key performance metric.

Based on data for stroke patients from the Belfast City Hospital, various scenarios have been explored with a focus on modelling phases which represent different stages of severity of illness and transition rates which are functions of important covariates, in this case age. The admission phase is characterised by the type of stroke, where different types of strokes have corresponding severity of illness and outcome. The results demonstrate the relationship between phase of discharge and expected total LOS, including the impact on bed occupancy. By exploring such scenarios, the key mechanisms for delay can thus be explored and their impact assessed.

Our current framework represents initial work towards developing integrated models for stroke services, including both hospital and community care, with the aim of supporting integrated planning. However, we believe that it also has considerable potential to be extended to include more detailed and explicit models of stroke services that allow us to assess complex scenarios involving interactions between services. Also, our current analytic model has the advantage that the results are based on routinely available discharge data. Another important aspect of extending our current framework is to attach costs to various options within the model. For example, we would like to be able to answer questions such as: should additional resources be put into thrombolysis for patients immediately after they have suffered a stroke, or is it better to focus on rehabilitative services in the community? Stroke is an excellent paradigm example enabling modelling of a whole health and social care system. The experience gained and techniques learned are likely to be relevant to the health and care of older persons in general. Phase-type models have an important role in this work.

## References

[1] Barton M, McClean SI, Gillespie J, Garg L, Wilson D, Fullerton K (2012). Is it beneficial to increase the provision of thrombolysis- a discrete event simulation model. QJM: An Int J Med.

[2] Gunal MM, Pidd M (2010). Discrete event simulation for performance modelling in health care: a review of the literature. J Simul.

[3] Dobson AJ, Barnett AG (2008). An introduction to generalized linear models.

[4] Fackrell M (2009). Modelling healthcare systems with phase-type distributions. Health Care Manag Sci.

[5] Faddy MJ, McClean SI (2000). Analysing data on lengths of stay of hospital patients using phase-type distributions. Appl Stoch Models and Data Anal.

[6] Faddy MJ, McClean SI (2005). Markov chain modeling for geriatric patient care. Meth Inform Med.

[7] Garg L, McClean S, Barton M, Meenan BJ, Fullerton K (2012). Intelligent patient management and resource planning for complex, heterogeneous, and stochastic healthcare systems. IEEE Trans Syst Man Cybern Syst Hum.

[8] Gillespie J, McClean S, Scotney B, Garg L, Barton M, Fullerton K (2011). Costing hospital resources for stroke patients using phase-type models. Health Care Management Science.

[9] Harper PR, Knight VA, Marshall AH (2012). Discrete Conditional Phase-type models utilising classification trees: application to modelling health service capacities. Eur J Oper Res.

[10] Harrison GW, Millard PH (1991). Balancing Acute and long-stay care. The mathematics of throughput in departments of geriatric medicine. Methods Inf Med.

[11] McGrory CA, Pettitt AN, Faddy M (2009). A fully Bayesian approach to inference for Coxian phase-type distributions with covariate dependent mean. Comput Stat Data Anal.

[12] Marshall AH, McClean SI (2003). Conditional phase-type distributions for modeling patient LOS in hospital. Intern Trans Oper Res.

[13] Marshall AH, McClean SI (2004). Using Coxian phase-type distributions to identify patient characteristics for duration of stay in hospital. Health Care Manag Sci.

[14] McClean SI, Millard PH (2007). Where to treat the older patient? Can Markov models help us better understand the relationship between hospital and community care. J Oper Res Soc.

[15] Millard PH, McClean SI (eds.) (1994) Modelling hospital resource use: a different approach to the planning and control of health care systems. Royal Society of Medicine Press

[16] McClean SI, Barton M, Garg L, Fullerton K (2011). A modeling framework that combines Markov models and discrete-event simulation for stroke patient care. ACM Transactions on Modelling and Computer Simulation (TOMACS).

[17] McClean S, Gillespie J, Garg L, Barton M, Scotney B, Fullerton K (2014). Using phase-type models to cost stroke patient care across health, social and community services. Eur J Oper Res.

[18] Millard PH (1991). Throughput in a department of geriatric medicine: a problem of time, space and behaviour. Health Trends.

[19] Neuts MF (1981). Matrix-geometric solutions in Stochastic Models: an algortihmic approach.

[20] Shahani AK, Ridley SA, Nielsen MS (2008). Modelling patient flows as an aid to decision-making for critical care capacities and organisation. Anaesthesia.

[21] Sundburg G, Bagust A, Terent A (2003). A model for costs of stroke service. Health Policy.

[22] Vasilakis C, Marshall AH (2005). Modelling nationwide hospital length of stay: Opening the black box. J Opl Res Soc.

[23] Xie H, Chaussalet TJ, Millard PH (2005). A continuous-time Markov model for the LOS of elderly people in institutional long-term care. J Royal Stat Soc Series A Statistics in Society.

